# Ac2-26 Alleviates Brain Injury after Cardiac Arrest and Cardiopulmonary Resuscitation in Rats via the eNOS Pathway

**DOI:** 10.1155/2020/3649613

**Published:** 2020-08-18

**Authors:** Jing Gong, Qi-Hang Tai, Guang-Xiao Xu, Xue-Ting Wang, Jing-Li Zhu, Xiao-Qing Zhao, Hai-Bin Sun, Dan Zhu, Wei Gao

**Affiliations:** ^1^Department of Anesthesiology, Chengdu Second People's Hospital, No. 10 Qingyun South Street, Jinjiang District, Chengdu 610021, China; ^2^Department of Anesthesiology, The Second Affiliated Hospital of Harbin Medical University, 246 Xuefu Road, Nangang District, Harbin 150000, China; ^3^Department of Neurology, The Second Affiliated Hospital of Harbin Medical University, 246 Xuefu Road, Nangang District, Harbin 150000, China

## Abstract

**Background:**

Brain injury is the leading cause of death following cardiac arrest (CA) and cardiopulmonary resuscitation (CPR). Ac2-26 and endothelial nitric oxide synthase (eNOS) have been shown to reduce neuroinflammation. This study is aimed at determining the mechanism by which Ac2-26 protects against inflammation during brain injury following CA and CPR.

**Methods:**

Sixty-four rats were randomized into sham, saline, Ac2-26, and Ac2-26+L-NIO (endothelial nitric oxide synthase (eNOS) inhibitor) groups. Rats received Ac2-26, Ac2-26+L-NIO, or saline after CPR. Neurologic function was assessed at baseline, 24, and 72 hours after CPR. At 72 hours after resuscitation, serum and brain tissues were collected.

**Results:**

Blood-brain barrier (BBB) permeability increased, and the number of surviving neurons and neurological function decreased in the saline group compared to the sham group. Anti-inflammatory and proinflammatory factors, neuron-specific enolase (NSE) levels, and the expression of eNOS, phosphorylated (p)-eNOS, inducible nitric oxide synthase (iNOS), and oxidative stress-related factors in the three CA groups significantly increased (*P* < 0.05). BBB permeability decreased, and the number of surviving neurons and neurological function increased in the Ac2-26 group compared to the saline group (*P* < 0.05). Ac2-26 increased anti-inflammatory and reduced proinflammatory markers, raised NSE levels, increased the expression of eNOS and p-eNOS, and reduced the expression of iNOS and oxidative stress-related factors compared to the saline group (*P* < 0.05). The effect of Ac2-26 on brain injury was reversed by L-NIO (*P* < 0.05).

**Conclusions:**

Ac2-26 reduced brain injury after CPR by inhibiting oxidative stress and neuroinflammation and protecting the BBB. The therapeutic effect of Ac2-26 on brain injury was largely dependent on the eNOS pathway.

## 1. Introduction

Cardiac arrest (CA) is a leading cause of morbidity and mortality worldwide [1]. Brain injury is the main cause of death in patients with spontaneous circulation recovery after CA [1]. Although cardiopulmonary resuscitation (CPR) techniques have improved, 30-50% of CA survivors suffer from cognitive impairment, and about 20% have a decline in quality of life [2]. The major pathological mechanisms underlying brain injury in CA include postischemic neurodegeneration, neuroinflammation, systemic inflammation, neurovascular injury, and cerebral edema [3].

AnnexinA1 (AnxA1) is an important endogenous mediator that induces the anti-inflammatory effect of glucocorticoids. AnxA1 is widely expressed in brain tissue [4–6]. Ac2-26, which is an exogenous active peptide of AnxA1, has been found to play a neuroprotective role. Specifically, Ac2-26 has been shown to protect against cerebral injury caused by stroke and is associated with decreased expression of cell adhesion and inflammatory factors [7]. Ac2-26 also plays a protective role in cerebral vascular injury during cerebral ischemia reperfusion [5]. Previous studies have also demonstrated that downregulation of endothelial nitric oxide synthase (eNOS) promotes the production of inflammatory cytokines and increases intercellular adhesion, thereby promoting inflammation [8]. Therefore, eNOS likely plays a key role in regulating inflammation during brain injury after CA. Based on these findings, we hypothesized that Ac2-26 could activate eNOS and ameliorate brain injury following CA and CPR. To test our hypothesis, we investigated the relationship between Ac2-26 and the eNOS pathway using the relatively specific eNOS inhibitor L-NIO [9, 10]. We found that Ac2-26-mediated protection against brain injury after CA and CPR was partially dependent on eNOS activation.

## 2. Materials and Methods

This study was approved by the Committee for Animal Protection and Utilization of the Second Affiliated Hospital of Harbin Medical University. This study was carried out in accordance with the national guidelines for animal studies and was approved by the Institutional Animal Care and Use Committee of the Second Affiliated Hospital of Harbin Medical University.

### 2.1. Animals

Male Sprague-Dawley rats (250-280 g) were obtained from the Animal Center of the Second Affiliated Hospital of Harbin Medical University.

### 2.2. CA and CPR

Rats were subjected to asphyxial CA and CPR according to previously published methods with minor modifications [11]. Briefly, all rats were anesthetized with 5% (*v*/*v*) sevoflurane, intubated, and then mechanically ventilated. Anesthesia was maintained with 1.5% (*v*/*v*) sevoflurane. The right femoral artery and vein were cannulated to monitor blood pressure and to inject medicines, respectively. Electrocardiograms were monitored and recorded using subcutaneous needle electrodes. After injection of vecuronium (0.2 mg/kg), asphyxia was induced by stopping mechanical ventilation. CA was deemed successful when the mean arterial pressure (MAP) was <25 mmHg.

After 8 minutes of asphyxia, CPR was started by performing chest compressions at a frequency of 60 breaths/min and ventilation of 1 ml/kg with 100% O_2_. Epinephrine (0.02 mg/kg) and 5% sodium bicarbonate (1 mmol/kg) were injected into the femoral vein at the initiation of chest compression. Restoration of spontaneous circulation (ROSC) was defined by heart rhythm restoration and a MAP >60 mmHg. Rats that received CPR for more than 3 minutes were excluded from this study. When the rats recovered spontaneous respiration, the mechanical ventilation was withdrawn and all catheters were removed. The rats were kept in air-filled boxes at room temperature (~25°C) and had free access to water and food. After 72 hours of ROSC, all rats were examined and sacrificed.

### 2.3. Animal Grouping

Sixty-four rats were randomized into sham, saline, Ac2-26, and Ac2-26+L-NIO groups (*n* = 16 per group). Rats in the sham group only received anesthesia and ventilation. All other rats received CA and CPR and then were injected with either saline, Ac2-26 (1 mg/kg) (Tocris Bioscience, Bristol, England), or Ac2-26+L-NIO (10 mg/kg) (Santa Cruz Biotechnology) after ROSC [10, 12].

### 2.4. Physiological Parameter Monitoring

Arterial blood gas was analyzed at baseline (defined as 10 minutes before the onset of asphyxia), 1, and 72 hours after ROSC to test pH, PaO_2_, PaCO_2_, K^+^, Na^+^, and Cl^−^ in the arterial blood. MAP measurements were recorded simultaneously.

### 2.5. BBB Permeability

#### 2.5.1. Brain Water Content

After 72 hours of ROSC, part of the brain tissue was collected and weighed (wet weight) and then dried at 60°C for 72 hours. Water content in the brain tissue was calculated as (wet weight − dry weight)/wet weight.

#### 2.5.2. Evans Blue Dye

Rats in each group were injected with a 2% Evans blue dye solution in saline (4 ml/kg). The dye was allowed to circulate for 2 hours. Cardiac perfusion with phosphate buffered saline (PBS) was performed under deep anesthesia. The brains were then removed, weighed, and homogenized in N, N-dimethylformamide (DMF). The homogenate was maintained at room temperature in the dark for 72 hours and centrifuged at 10,000 × g for 25 minutes. Absorbance of the supernatant was measured using a spectrophotometer (Thermo Spectronic Genesys 10 UV, Thermo Fischer Scientific Inc., Waltham, MA, USA) at 610 nm [13]. The amount of Evans blue dye was calculated using a standard curve and is expressed as microgram/gram brain tissue.

### 2.6. Histopathological Analysis

The brain tissues of 8 rats in each group were perfused and fixed with 4% paraformaldehyde. The tissues were sectioned into 30-mm-thick slices and mounted on polylysine-coated slides overnight. On the next day, the sections were rehydrated in distilled water and submerged in 1% cresyl violet for 10 minutes until the desired depth of staining was achieved. Nissl staining was performed to determine neuronal survival. The number of surviving neurons in the hippocampal CA1 region was observed and recorded in three separate nonadjacent sections from each animal, using the ImageJ software. Neurons with clear characteristics of a visible nucleus and intact cytoplasm were considered to be surviving neurons. Neuronal staining evaluation was performed by a pathologist who was blinded to the group assignment.

### 2.7. Evaluation of Neurologic Function

Neurologic function was determined using neurological deficit scores (NDS) at baseline, 24, and 72 hours after ROSC. The evaluation of NDS included 7 aspects: general behavior, brain stem function, motor assessment, sensory assessment, motor behavior, behavior, and seizures [11] (Table 1). Functional assessment was performed by two independent researchers. An NDS of 80 represents normal brain function, and an NDS of 0 represented neuronal death.

### 2.8. Cytokine Detection

At 72 hours after ROSC, brain tissues and serum were collected and homogenized with a tissue diluent to prepare the homogenate. After centrifugation, the supernatant was collected. To evaluate the effect of Ac2-26 on local inflammation, we measured the concentrations of IL-1*β*, IL-6, and IL-10 in the homogenate and NSE levels in the serum using enzyme linked immunosorbent assays (ELISA) (Nanjing Jiancheng Corp, China).

### 2.9. Western Blot

Brain tissues were collected and lysed in RIPA buffer in a mixture containing protease and phosphatase inhibitors (Beyotime Biotechnology, Jiangsu, China). Protein concentrations in each sample were calculated using the Bradford assay. Equal amounts of total protein from the tissue lysates were separated using sodium dodecyl sulfate polyacrylamide gel electrophoresis (SDS-PAGE) and then transferred to polyvinylidene fluoride membranes (PVDF, Roche). The membranes were blocked in 5% dry milk (diluted with Tris buffered saline with Tween 20 (TBST) buffer) for 2 hours at room temperature and then incubated with rabbit polyclonal anti-iNOS, eNOS, p-eNOS (1 : 1,000; Cell Signaling Technology, USA), p-NF-*κ*B (1 : 1000; ABclonal, China), and mouse monoclonal anti-*β*-actin (1 : 3,000; Boster Biological Technology, China) primary antibodies (diluted in 1X TBST with 5% bovine serum albumin (BSA)) for 16 hours at 4°C. The membranes were washed three times for 5 minutes with TBST at room temperature and incubated with a secondary horseradish peroxidase-conjugated sheep anti-rabbit or anti-mouse IgG antibody (1 : 5,000; Boster Biological Technology, CHINA) for 1 hour at room temperature. After washing three times for 5 minutes with TBST at room temperature, the protein bands were detected using horseradish peroxidase (Santa Cruz Biotechnology) and visualized using enhanced chemiluminescence.

### 2.10. Oxidative Stress Response

Malondialdehyde (MDA) levels and myeloperoxidase (MPO) and xanthine oxidase (XO) activation in brain tissues were analyzed using a Coomassie blue dye-binding assay (Nanjing Jiancheng Corp, China) according to the manufacturer's instructions.

### 2.11. Statistical Analysis

All data are presented as the mean ± standard deviation (SD). Differences among groups were analyzed using analysis of a one-way analysis of variance. All analyses were conducted using SPSS software version 19.0 (SPSS, Chicago, IL, USA). A *P* value < 0.05 was considered statistically significant.

## 3. Results

Two rats in the saline group, one rat in the Ac2-26 group, and one rat in the Ac2-26+L-NIO group failed to be resuscitated within 3 minutes of CPR and were excluded from all analyses.

### 3.1. Physiologic Variables

There were no significant differences in body weight and temperature between the groups (data not shown). Parameters related to the resuscitation procedure, including mean arterial pressure (MAP), pH, partial pressure of oxygen (PaO_2_), partial pressure of carbon dioxide (PaCO_2_), K^+^, Na^+^, and Cl^−^, did not differ in the three CA groups.

### 3.2. Ac2-26 Ameliorated Blood-Brain Barrier (BBB) Permeability

**Figure 1:**
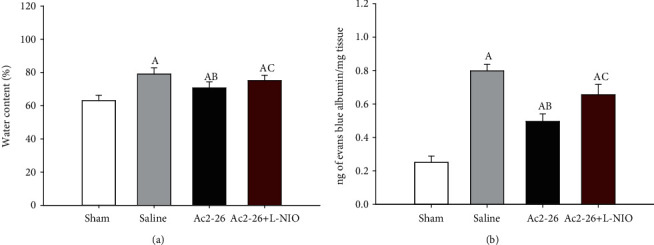
BBB integrity after ROSC. The wet/dry ratio (a) and levels of Evan's blue dye (b) in the brain tissues were measured 72 hours after ROSC. Data are presented as mean ± SD. ^A^*P* < 0.05 vs. sham group; ^AB^*P* < 0.05 vs. saline group; ^AC^*P* < 0.05 vs. Ac2-26 group (, sham group; , saline group; , Ac2-26 group; , Ac2-26+L-NIO group).

We did not observe brain edema in the sham group; however, BBB permeability deteriorated in rats following restoration of spontaneous circulation (ROSC). The wet/dry ratio and Evans blue dye in the brain tissues were significantly increased in the saline group compared to the Ac2-26 group. The eNOS inhibitor L-NIO reduced the effects of Ac2-26 on BBB (*P* < 0.05) (Figure 1).

### 3.3. Ac2-26 Increased Neuronal Survival

**Figure 2:**
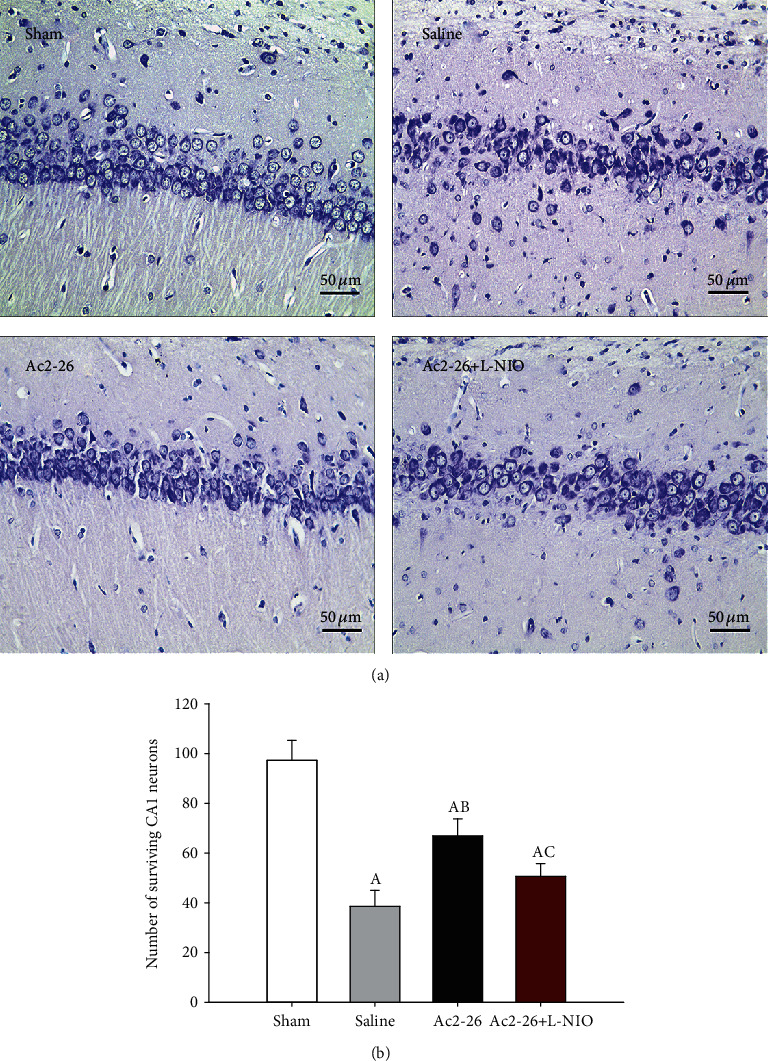
Number of surviving CA1 neurons after ROSC. Nissl staining was used to measure neuronal survival in the hippocampal CA1 region 72 hours after ROSC (magnification 400-fold) (a) The number of surviving neurons in the CA1 region of the hippocampus. (b) Data are presented as mean ± SD. ^A^*P* < 0.05 vs. sham group; ^AB^*P* < 0.05 vs. saline group; ^AC^*P* < 0.05 vs. Ac2-26 group (, sham group; , saline group; , Ac2-26 group; , Ac2-26+L-NIO group).

We observed a decrease in neuronal survival in the hippocampal CA1 region of the brain in the three CA groups compared to the sham group (*P* < 0.05). The number of surviving hippocampal CA1 neurons in the Ac2-26 group was significantly higher compared to the saline group (*P* < 0.05). However, L-NIO significantly reversed the protective effect of Ac2-26 on neuronal survival (*P* < 0.05) (Figure 2).

### 3.4. Ac2-26 Improved the Neurological Deficit Scores (NDS)

**Figure 3:**
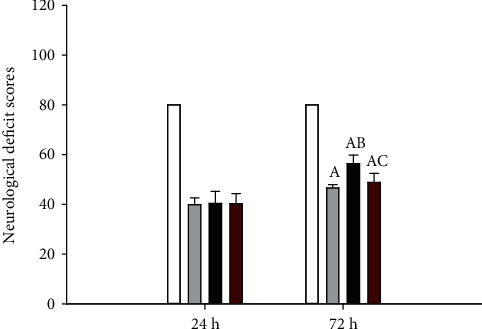
NDS after ROSC. The NDS were measured by evaluating the behavior of the rats at 24 and 72 hours after ROSC. Data are presented as mean ± SD. ^A^*P* < 0.05 vs. sham group; ^AB^*P* < 0.05 vs. saline group; ^AC^*P* < 0.05 vs. Ac2-26 group (, sham group; , saline group; , Ac2-26 group; , Ac2-26+L-NIO group).

The NDS of all four groups was 80 at baseline (data not shown). However, following CA, the NDS significantly decreased 24 h after ROSC, and there were no significant differences among the three CA groups with regard to neurological status at this time point. The rats that underwent CA showed motor incoordination, sensory disturbance, spastic paralysis of hind limbs, seizures, and other neurological damage presentation, compared to the sham group. The NDS of rats in the saline group were obviously lower compared to those in the sham group (*P* < 0.05). After 72 h, the NDS of the rats in the Ac2-26 group were significantly higher compared to those in the saline group (*P* < 0.05). However, L-NIO partially reversed the effect of Ac2-26 on NDS (*P* < 0.05) (Figure 3).

### 3.5. Ac2-26 Reduced Brain Damage

**Figure 4:**
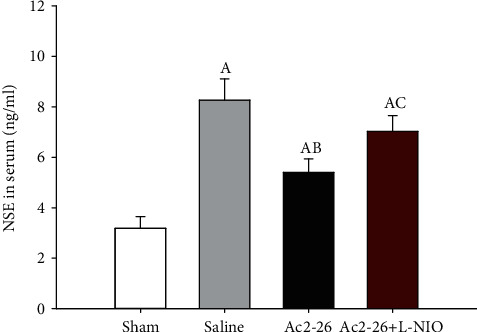
Serum NSE levels after ROSC. Serum NSE levels were detected using ELISA 72 hours after ROSC. Data are presented as mean ± SD. ^A^*P* < 0.05 vs. sham group; ^AB^*P* < 0.05 vs. saline group; ^AC^*P* < 0.05 vs. Ac2-26 group (, sham group; , saline group; , Ac2-26 group; , Ac2-26+L-NIO group).

Neuron-specific enolase (NSE) serum levels were measured 72 hours after resuscitation. NSE levels in nerve tissues have been reported to be closely related to the prognosis of neurological function in ischemic brain injury [14]. NSE levels significantly increased in the three CA groups compared to those in the sham group (*P* < 0.05). Ac2-26 decreased the NSE levels (*P* < 0.05), and L-NIO treatment reversed the effects of Ac2-26 (*P* < 0.05) (Figure 4).

### 3.6. Ac2-26 Reduced Neuroinflammation

**Figure 5:**
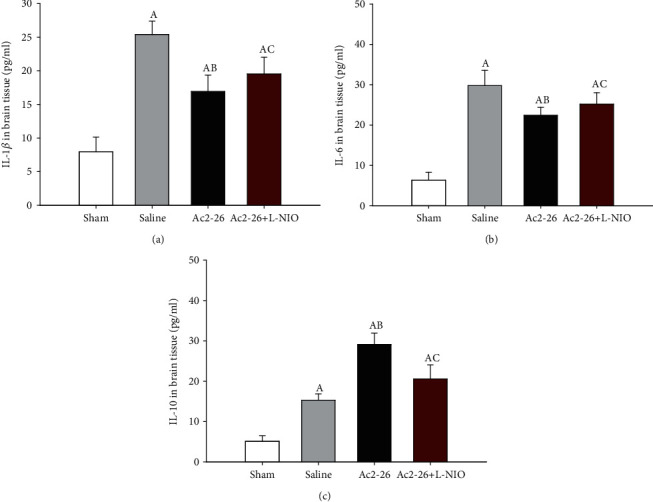
Cytokine (IL-1*β*, IL-6, and IL-10) expression in the brain tissues after ROSC. Cytokine (IL-1*β*, IL-6, and IL-10) expression in the brain tissue was measured using ELISA 72 hours after ROSC. Data are presented as mean ± SD. ^A^*P* < 0.05 vs. sham group; ^AB^*P* < 0.05 vs. saline group; ^AC^*P* < 0.05 vs. Ac2-26 group (, sham group; , saline group; , Ac2-26 group; , Ac2-26+L-NIO group).

**Figure 6:**
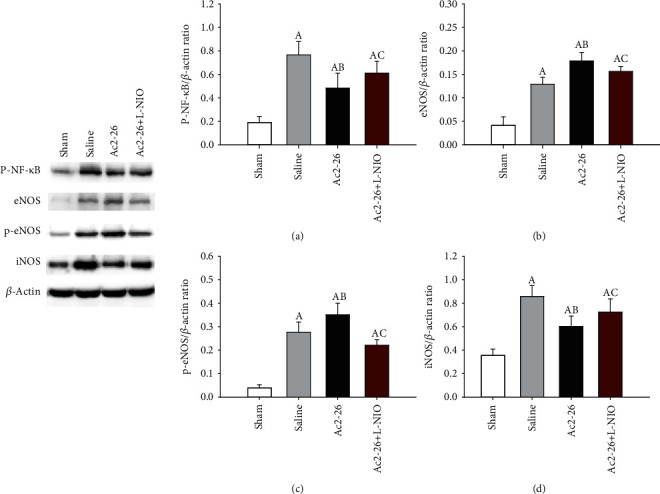
Expression of p-NF-*κ*B, eNOS, p-eNOS, and iNOS in brain tissue after ROSC. p-NF-*κ*B, eNOS, p-eNOS, and iNOS were measured using Western blot analysis 72 hours after ROSC. p-NF-*κ*B (a); eNOS (b); p-eNOS (c); and iNOS (d). Data are presented as mean ± SD. ^A^*P* < 0.05 vs. sham group; ^AB^*P* < 0.05 vs. saline group; ^AC^*P* < 0.05 vs. Ac2-26 group (, sham group; , saline group; , Ac2-26 group; , Ac2-26+L-NIO group).

We next measured the expression of cytokines ((interleukin (IL)-1*β*, IL-6, and IL-10) and (phosphorylated nuclear factor-*κ*B (p-NF-*κ*B))) in brain tissues. Compared to the sham group, the inflammatory cytokines IL-1*β* and IL-6 and p-NF-*κ*B significantly increased after CA. Compared to the saline group, IL-1*β* and IL-6 expression significantly decreased, and the anti-inflammatory cytokine IL-10 significantly increased in the Ac2-26 group (*P* < 0.05). However, compared to the Ac2-26 group, L-NIO significantly increased IL-1*β* and IL-6 expression, but decreased IL-10 expression, in the Ac2-26+L-NIO group (*P* < 0.05) (Figure 5). Similarly, p-NF-*κ*B expression in the brain tissues was significantly upregulated in rats following CA. Compared with the saline group, Ac2-26 significantly reduced p-NF-*κ*B expression, but this reduction was significantly reversed by L-NIO (*P* < 0.05) (Figure 6).

### 3.7. eNOS, P-eNOS, and iNOS Expression

After CA and CPB, the levels of eNOS, p-eNOS, and iNOS were significantly upregulated in the brain tissues. Compared to the saline group, eNOS and p-eNOS expression was significantly increased, and iNOS expression was reduced (*P* < 0.05) in the Ac2-26 group. Compared to the Ac2-26 group, L-NIO significantly inhibited eNOS and p-eNOS expression but enhanced iNOS expression in the brain tissues (*P* < 0.05) (Figure 6).

### 3.8. Ac2-26 Inhibited Oxidative Stress

**Figure 7:**
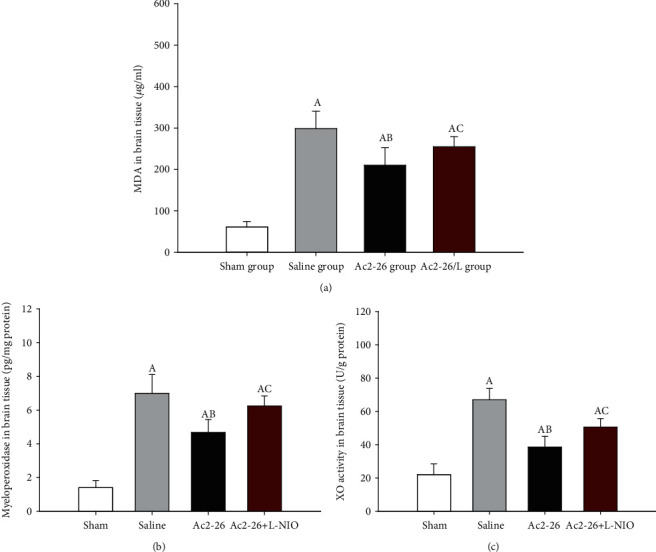
Oxidative stress related factors in the brain tissue after ROSC. MDA levels and MPO and XO activation were detected in the brain tissues. Data are presented as mean ± SD. ^A^*P* < 0.05 vs. sham group; ^AB^*P* < 0.05 vs. saline group; ^AC^*P* < 0.05 vs. Ac2-26 group (, sham group; , saline group; , Ac2-26 group; , Ac2-26+L-NIO group).

After 72 h of resuscitation, we measured MDA levels and MPO and XO activation in the brain tissues. After CA and CPR, we observed a severe oxidative stress response (*P* < 0.05). In the Ac2-26 group, MDA levels and MPO and XO activation were significantly decreased relative to the saline group (*P* < 0.05). L-NIO significantly inhibited the effects of Ac2-26 on oxidative stress (*P* < 0.05) (Figure 7).

## 4. Discussion

In this study, we demonstrated that the active peptide of AnxA1 and Ac2-26, significantly reduced the brain injury induced by CA, improved neurological function and BBB permeability, and decreased neuroinflammation and oxidative stress. We also showed that the protection conferred by Ac2-26 was partially dependent on the eNOS pathway.

Anoxic-ischemic brain injury is the leading cause of mortality after CA and CPR. Following CA, the heart stops pumping completely. Subsequently, cerebral oxygen stores are lost within 20 seconds, and glucose and adenosine triphosphate are depleted within 5 minutes [1]. Lack of cellular ATP leads to dysfunction of ion pumps and accumulation of Na^+^ and Ca^2+^ in the cells, which promotes the release of excitatory neurotransmitters. Consequently, N-methyl D-aspartate-regulated (NMDA) cation channels are activated, and more Ca^2+^ enters the cells, leading to the production of reactive oxygen species (ROS) and the formation of hypoxanthine [15]. During tissue reperfusion/reoxidation, XO converts hypoxanthine to uric acid to form superoxides [16]. During CA, hypoxia leads to ROS production and secondary local inflammation, which further results in brain damage, increased BBB permeability, neuron apoptosis, and neurological dysfunction [1, 17, 18].

AnxA1 and its active peptide, Ac2-26, have been suggested to reduce heart and lung ischemia reperfusion injury [12, 19]. We previously reported that Ac2-26 can reduce lung ischemia reperfusion injury by regulating inflammation [20]. Exogenous AnxA1 administration can inhibit the noninflammatory phagocytosis of apoptotic neurons and reduce the production of inflammatory mediators [21]. Ac2-26 was found to reduce cerebral edema in rats after ROSC and reduce the distribution of Evans blue staining in brain tissue. We found that Ac2-26 reduced BBB permeability and thus increased neuronal survival and improved nerve function. NSE overexpression is commonly used as a specific marker of brain injury severity [22]. In this study, we found that Ac2-26 decreased NSE levels after ROSC. We speculate that the protection conferred by Ac2-26 may be associated with its antioxidative and anti-inflammatory properties.

XO is a key enzyme in purine catabolism and a major source of free radicals [23]. During CA, anaerobic metabolism will generate ROS in response to XO production. The increase in ROS leads to lipid peroxidation, with MDA as the final product of this process. As such, MDA is an effective biomarker of lipid oxidation [24]. MPO can be released by activated microglia and astrocytes, resulting in an increase in highly reactive oxidants and free radicals that are harmful to brain function [25]. In contrast, NO reacts with superoxide to form peroxynitrite (ONOO^−^), which also plays a role during brain injury [26]. Excessive ROS activate iNOS to produce NO [26]. A deficiency in eNOS also contributes to enhanced NO generation via the nitrate-nitrite-NO pathway [27]. In this study, we found that Ac2-26 significantly reduced MDA levels and inhibited XO and MPO activation after CA and CPR. These results indicate that Ac2-26-mediated amelioration of brain injury may be due to inhibition of oxidative stress. Ac2-26 significantly reduced iNOS and increased eNOS expression after CA and CPR. Therefore, we speculate that the therapeutic effect of Ac2-26 after CA and CPR may be achieved by modulating iNOS and eNOS levels.

During ischemia and hypoxia, NF-*κ*B is rapidly activated in response to increases in oxidative stress, which promotes the production of inflammatory factors [28]. In this study, we found that Ac2-26 significantly reduced inflammatory cytokine (IL-1*β* and IL-6) levels and that the Ac2-26-mediated effects on local inflammation were associated with NF-*κ*B inhibition. Cell death and release of excitatory toxic molecules cause microglia to secrete cytokines into the brain, further aggravating brain injury [1]. Ac2-26 can promote the transformation of microglial cells from the proinflammatory M1 status to the anti-inflammatory M2 status, which results in a reduction of inflammatory cytokines and protection of neurons [6]. Microglial cells also express IL-10, which is as an anti-inflammatory cytokine [29]. Since Ac2-26 reduced proinflammatory factors and increased anti-inflammatory factors in the brain tissues, Ac2-26 likely reduces local inflammation by regulating NF-*κ*B activation in microglia cells.

Ac2-26 has been shown to activate the PI3K/AKT pathway [19]. Phosphorylation of upstream AKT promotes eNOS activation [30]. Therefore, to explore the possible mechanism of Ac2-26 in brain injury after ROSC, we administrated L-NIO, an eNOS inhibitor, to determine if Ac2-26-mediated protection is dependent on eNOS. L-NIO significantly reversed the effects of Ac2-26 on neurological function, histological score, and edema. Local oxidative stress and inflammation were also increased following L-NIO treatment. These results suggest that the protection conferred by Ac2-26 on brain injury after CA is partially dependent on eNOS activation.

## 5. Conclusion

Ac2-26 reduced brain injury after CPR by inhibiting oxidative stress and neuroinflammation and protecting the BBB. The therapeutic effect of Ac2-26 on brain injury was largely dependent on the eNOS pathway.

## 6. Limitations

There are several limitations in our study. First L-NIO is a nonspecific eNOS inhibitor. In our future study, we will use microRNA to interfere with AKT activation to further explore the mechanism by which Ac2-26 protects against brain injury after CA. Second, the present study did not measure the effect of Ac2-26 on cell apoptosis in brain tissue. It has been shown that Ac2-26 can enhance apoptosis of inflammatory cells, such as neutrophils [31]. Our future study will utilize a cell model to investigate the effect of Ac2-26 on cell apoptosis in brain tissue.

## Figures and Tables

**Table 1:** Neurological deficit scores (NDS) for rats.

General behavior	Total score = 19
Consciousness	Normal (10), stuporous (5), comatose (0)
Arousal	Eyes open spontaneously (3), eyes open to pain (1), no eye opening (0)
Respiration	Normal (6), abnormal (0), absent (0)
Brain-stem function	Total score = 21
Olfaction	Present (3), absent (0)
Vision	Present (3), absent (0)
Pupillary reflex	Present (3), absent (0)
Corneal reflex	Present (3), absent (0)
Startle reflex	Present (3), absent (0)
Whisker stimulation	Present (3), absent (0)
Swallowing	Present (3), absent (0)
Motor assessment	Total score = 6
Strength (left and right side tested and scored separately)	Normal (3), stiff/weak (1), no movement/paralyzed (0)
Sensory assessment	Total score = 6
Pain (left and right side tested and scored separately)	Brisk withdrawal with pain (3), weak or abnormal response (1), no withdrawal (0)
Motor behavior	Total score = 6
Gait coordination	Normal (3), abnormal (1), absent (0)
Balance on beam	Normal (3), abnormal (1), absent (0)
Behavior	Total score = 12
Righting reflex	Normal (3), abnormal (1), absent (0)
Negative geotaxis	Normal (3), abnormal (1), absent (0)
Visual placing	Normal (3), abnormal (1), absent (0)
Turning alley	Normal (3), abnormal (1), absent (0)
Seizures	Total score = 10
Convulsive or nonconvulsive	No seizure (10), focal seizure (5), general seizure (0)

## Data Availability

The data used to support the findings of this study are available from the corresponding author upon request.
